# Association of sarcopenia with generalized anxiety disorder in Korean middle-aged and older adults: Results from the Korea National Health and Nutrition Examination Survey in 2022

**DOI:** 10.1371/journal.pone.0323772

**Published:** 2025-05-15

**Authors:** Hye-Eun An, Hye-Su Park, Chang Shik Yin

**Affiliations:** 1 Department of East-West Medicine, Kyung Hee University, Yongin, Republic of Korea; 2 Department of Medical Education, College of Korean Medicine, Kyung Hee University, Seoul, Republic of Korea; Sengkang General Hospital, SINGAPORE

## Abstract

We highlighted sarcopenia’s association with psychiatric disorders, including generalized anxiety disorder (GAD), and aimed to explore correlations between the detection and prevention of GAD using population-based data of middle-aged and older Korean adults. This cross-sectional study data was collected through standardized surveys and physical examinations including GAD assessment survey and sarcopenia examination through muscle strength and mass assessments. We used logistic regression analysis to examine the association between sarcopenia and GAD, adjusting for relevant confounders. Data from the 2022 Korea National Health and Nutrition Examination Survey (KNHANES) were analyzed for sarcopenia and GAD in individuals aged ≥40 years, employing the 2019 Asian Working Group for Sarcopenia criteria and GAD-7 scale, respectively. Overall, 2960 individuals were selected, focusing on sarcopenia and GAD-7 scores, and regression analyses explored sarcopenia and GAD associations. Notably, 3.7% and 14% of participants had sarcopenia and GAD, respectively. The relationships between sarcopenia, possible sarcopenia, and GAD were found for females, suggesting sex-specific risks. In females, after adjusting for marital status and smoking, sarcopenia and possible sarcopenia showed correlation coefficients (β) of 0.145 (95% confidence interval [CI]: 0.025–0.266) and 0.096 (95% CI: 0.004–0.189), respectively, with GAD. After further adjustment for physical activity variables, only sarcopenia remained significantly associated with GAD in females (β=0.132, P = 0.033). Sarcopenia was significantly associated with GAD severity. Individuals with sarcopenia were 1.051 times more likely to experience mild GAD (95% CI: 0.554–1.992) and 2.480 times more likely to experience moderate to severe GAD (95% CI: 1.232–4.990) compared to those without sarcopenia. This study found a significant correlation between sarcopenia and GAD in the examined demographics, emphasizing the importance of integrative physical and mental health interventions. Early detection and management of sarcopenia may contribute to GAD management, advocating a holistic approach to healthcare in aging populations.

## Introduction

Aging is a natural phenomenon that leads to various physiological alterations [1]. Sarcopenia, characterized by the progressive loss of skeletal muscle mass and strength, is primarily associated with aging [2]. It has been linked to several adverse health outcomes, including fractures, dependency, and increased mortality risk [3]. Additionally, evidence indicates that sarcopenia correlates with neurological and psychiatric disorders such as dementia, cognitive decline, and depression [4]. Research on sarcopenia has traditionally focused on older adults. However, recent findings indicate that its precursors may develop earlier in life, highlighting the need to study sarcopenia across different stages of adulthood for early detection and prevention [5,6].

Sarcopenia has been widely studied in oncologic settings, particularly in relation to its prognostic significance and impact on treatment outcomes among cancer patients [7]. While its role in post-treatment rehabilitation and psychological well-being has been suggested, its impact on psychiatric disorders, such as generalized anxiety disorder (GAD), remains underexplored despite its high prevalence in these populations [8]. Generalized anxiety disorder (GAD) is a widespread mental health condition characterized by excessive and uncontrollable worry about various aspects of daily life. Studies have demonstrated its significant association with physical health conditions, particularly in individuals with severe mental illness [9]. Individuals with GAD frequently experience musculoskeletal disorders and other physical health conditions [10]. However, the relationship between sarcopenia and GAD remains unclear.

Notably, several mechanisms have been proposed to explain the association between sarcopenia and GAD. Chronic inflammation significantly contributes to the progression of sarcopenia and the onset of anxiety disorders [11]. Excessive oxidative stress is another critical factor linking sarcopenia to anxiety disorders, leading to low muscle mass and various psychiatric conditions, including GAD [12]. Moreover, Brain-Derived Neurotrophic Factor (BDNF), generated during skeletal muscle contractions, has been recognized for its impact on the onset of anxiety disorders [13,14]. Finally, inadequate physical activity and poor nutritional intake are common risk factors for sarcopenia and GAD [15].

Despite the increasing awareness of the connection between mental and physical health, the relationship between sarcopenia and GAD remains inconclusive. Some studies have found no correlation between muscle function and GAD [16], whereas others have identified a significant link between muscle strength and GAD [17]. These discrepancies may arise from the different criteria used to diagnose sarcopenia and GAD. However, the association of sarcopenia with GAD in the Korean population remains inconclusive. To address this gap, we used data from the Korea National Health and Nutrition Examination Survey (KNHANES) to explore the correlation between sarcopenia and GAD among middle-aged and older adults in Korea. Sarcopenia was assessed using the Asian Working Group for Sarcopenia (AWGS) 2019 diagnostic criteria, and GAD was evaluated using the GAD-7 scale.

This study is particularly significant as one of the first to investigate the correlation between sarcopenia and the severity of GAD using a large-scale, representative sample of the Korean population. There are currently no other studies focusing on this specific demographic, highlighting a critical gap in the existing literature. Understanding this relationship may lead to targeted interventions that address sarcopenia and GAD, ultimately improving overall health outcomes in this population.

This study provides real-world, population-based evidence on the association between physical and mental health by examining the link between sarcopenia and GAD. Identifying sarcopenia as a potential risk factor for GAD in middle-aged and older adults highlights the urgent need for early detection and targeted intervention strategies to reduce the burden of both conditions.

## Methods

### Study population

The Ministry of Health and Welfare has conducted the KNHANES, a cross-sectional survey to assess the health and nutrition of Koreans, annually since 1998. Data were collected from participants using standardized questionnaires on their health status, health behaviors, and dietary intake. Our study utilized data from the first year of the ninth cycle (2022 KNHANES), which also included results from appendicular muscle mass and grip strength tests.

**Fig 1:**
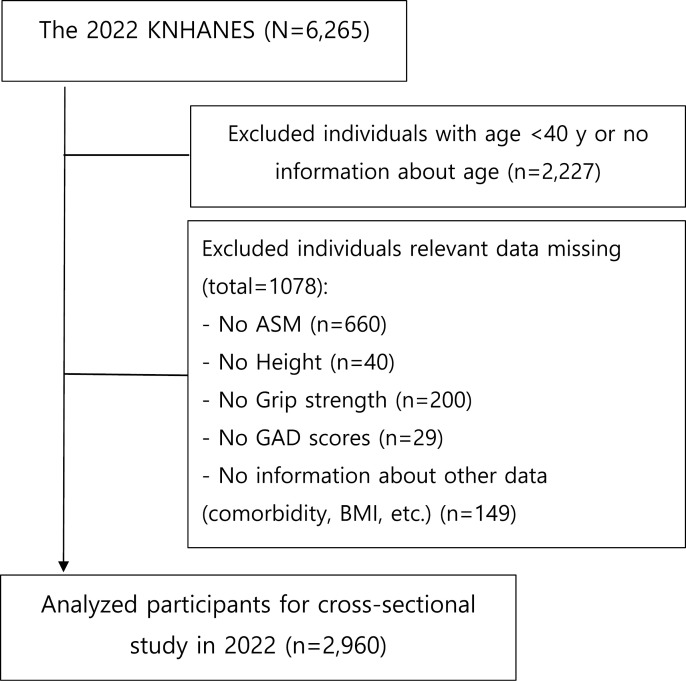
Flowchart for the study sample. KNHANES, Korea National Health and Nutrition Examination Survey; ASM, appendicular skeletal muscle mass; GAD-7, generalized anxiety disorder-7; BMI, body mass index.

This survey recruited 6,265 participants from 4,800 households across 192 regions. We included individuals based on the following criteria: (1) age ≥ 40 years; (2) available data on sarcopenia status, including appendicular muscle mass, grip strength, and height measurements; and (3) accessible GAD-7 scores. The age cutoff of 40 years was selected based on prior research indicating that sarcopenia may develop as early as midlife, with muscle loss accelerating beyond this age [2]. Additionally, GAD has been reported to become more prevalent and chronic in individuals over 40 [10]. AWGS 2019 also emphasizes the importance of early detection and intervention in middle-aged individuals who may be at risk of sarcopenia [18]. The exclusion criteria were: (1) lack of age data; (2) age < 40 years; (3) incomplete sarcopenia status data, including appendicular muscle mass, grip strength, and height measurements; (4) absence of GAD-7 scores; and (5) missing information on other relevant factors such as comorbid conditions and body mass index (BMI). We excluded 3,305 individuals based on these criteria. Specifically, 2,227 individuals were excluded because of missing age data or age < 40 years. Additionally, 660 participants were excluded owing to the absence of appendicular muscle mass data, 40 were excluded because of missing height data, and 200 were excluded due to missing grip strength test results. Furthermore, 29 individuals lacked GAD-7 scores, and 149 were excluded because they did not have supplementary information. Therefore, 2,960 participants were included in this study. Fig 1 illustrates the detailed screening process. The appropriate ethics committee process has been cleared for this study design (KHSIRB-24-207EA).

### Assessment of sarcopenia status

Sarcopenia was diagnosed based on the 2019 guidelines of the AWGS. These guidelines divide sarcopenia into three stages based on specific criteria: no sarcopenia, possible sarcopenia, and sarcopenia. Sarcopenia is diagnosed when there is evidence of low muscle strength and/or reduced physical performance; however, sarcopenia requires low muscle mass in addition to these factors. In Asia, most research concerning muscle strength and sarcopenia has considered handgrip strength as an indicator of overall muscle strength; therefore, the AWGS 2019 endorses the measurement of handgrip strength as a marker for muscle strength [18]. Participants with low muscle strength were classified as having possible sarcopenia, whereas those with low muscle strength and low muscle mass were classified as having sarcopenia.

#### Muscle strength.

A digital hand dynamometer (T.K.K 5401, Japan) was used to measure the grip strength of both hands twice, and the highest measurement was used to diagnose low muscle strength. In this study, low muscle strength was defined as < 28 kg for males and < 18 kg for females, based on the grip strength cut-off points presented in the AWGS 2019.

#### Muscle mass.

Muscle mass was measured using an impedance body fat meter (Inbody 970, Biospace, Korea), and appendicular skeletal muscle mass (ASM) was determined by adding the muscle masses of the arms and legs. The skeletal muscle mass index for sarcopenia was described as ASM per square of height (ASM [kg]/height [m²]). In this study, low muscle mass was defined as < 7 kg/m² and < 5.7 kg/m² for males and females, respectively, based on the muscle mass cut-off points presented in the AWGS 2019.

### Assessment of generalized anxiety disorder (GAD)

The 2022 KNHANES used the GAD-7 scale to evaluate GAD. The scale comprises seven items, each assessing (1) feeling nervous, anxious, or restless; (2) inability to cease or manage worrying; (3) excessive concern about various matters; (4) difficulty calming down; (5) restlessness or feeling keyed up or on edge; (6) quick irritation and agitation; and (7) experiencing a sense of fear as if something terrible may occur. Responses to these seven items were rated on a scale of “not at all’ (0 points),” several days’ (1 point), “more than half the days’ (2 points), and” nearly every day’ (3 points) based on how often they were experienced over the past 2 weeks. The scores for each item were summed, and the total scores ranging from 0–4 were categorized as normal, 5–9 as mild, 10–14 as moderate, and 15–21 as severe levels of GAD. However, in this study, we defined scores of 0–4 as normal, 5–9 as mild, and 10–21 as moderate/severe.

### Assessment of covariates

Covariates included sociodemographic and health-related factors. The sociodemographic variables included age, sex, marital status (married or unmarried), educational level (elementary school or below, middle school or high school, and college or above), and socioeconomic status. Household income was defined based on the quintile system into three socioeconomic groups (low: first quintile, middle: second to fourth quintiles, high: fifth quintile). Health-related factors considered included smoking status (current smokers versus former or never smokers), alcohol consumption advisories (those advised to quit or flagged by doctors or family versus those who were never advised), chewing issues (present or absent), physical activity frequency (active thrice or more weekly versus less active), BMI (underweight: BMI < 18.5 kg/m²; normal weight: BMI < 23.0 kg/m²; and overweight or obese: BMI ≥ 23.0 kg/m²), and diagnosed chronic conditions (hypertension, dyslipidemia, diabetes mellitus, asthma, sinusitis, allergic rhinitis, obstructive sleep apnea, kidney disease, atopic dermatitis, and otitis media). The physical activity level was assessed based on the weekly frequency of walking exercises or strength training and categorized as either active (three or more times weekly) or less active (fewer than three times weekly) [19].

### Statistical analysis

The KNHANES data involve complex sample analyses; therefore, all analyses were conducted using weighted variables. Continuous and categorical variables were represented using means ± standard errors and percentages, respectively. First, the basic features of the samples were analyzed using chi-squared tests, Student’s t-tests, and an analysis of variance. Second, the sample was separated into sarcopenia and possible sarcopenia categories, and a multiple linear regression analysis was used to investigate the correlation of each category with GAD. Finally, logistic regression analyses were conducted to examine the association of sarcopenia with the three GAD groups by calculating odds ratios and 95% confidence intervals (CIs). The models were adjusted for different sets of covariates. Model 1 included sex, marital status, and smoking status. Model 2 additionally controlled for physical activity, while Model 3 further adjusted for chewing problems, BMI, and chronic conditions. All statistical analyses were conducted using R statistical software (version 4.3.2; R Foundation for Statistical Computing, Vienna, Austria). Statistical significance in all tests was set at P < 0.05.

## Results

The basic features of the study samples based on sarcopenia status are summarized in Table 1. This study included 2,960 participants; 1297 males (43.8%) and 1,663 females (56.2%). The prevalence of sarcopenia in those aged ≥40 years was 3.7% (109/2,960). Sarcopenia was significantly associated with age, sex, chewing problems, physical activity, including walking and strength exercises, education level, household income, BMI, and hypertension.

**Table 1:** Features of study samples based on sarcopenia status (n = 2,960).

Variables	Overall (n = 2960)	Sarcopenia (n = 109)	No sarcopenia (n = 2851)	P-value
Age (years)	59.8 ± 11.6	73.3 ± 10	59.4 ± 11.4	<0.001^++^
Male	1,297 (43.8)	37 (33.9)	1,260 (44.2)	0.044^*^
Ever married (vs. others)	2,809 (94.9)	104 (95.4)	2,705 (94.9)	0.979
Currently smoker (vs. others)	439 (14.8)	11 (10.1)	428 (15.0)	0.200
Drinking problem	374 (12.6)	11 (10.1)	363 (12.7)	0.505
Chewing problem	1,092 (36.9)	57 (52.3)	1,035 (36.3)	< 0.001^++^
**Physical activity (per week)**				
Walking exercises	2,015 (68.1)	59 (54.1)	1,956 (68.6)	0.002^+^
Strength exercises	529 (17.9)	11 (10.1)	518 (18.2)	0.042^*^
**Education level**				<0.001^++^
Elementary school or below	544 (18.4)		491 (17.2)	
Middle or high school	1,279 (43.2)		1,243 (43.6)	
College or above	1,137 (38.4)		1,117 (39.2)	
**Household income (quintile)**				<0.001^++^
Upper (1st)	678 (22.9)		666 (23.4)	
Middle (2nd–4th)	1,820 (61.5)		1,771 (62.1)	
Lower (5th)	462 (15.6)		414 (14.5)	
**Body mass index category**				< 0.001^++^
Underweight	48 (1.6)	12 (11.0)	36 (1.3)	
Normal	1,101 (37.2)	65 (59.6)	1,036 (36.3)	
Overweight or obese	1,811 (61.2)	32 (29.4)	1,779 (62.4)	
**Comorbidities**				
Hypertension	1,000 (33.8)	55 (50.5)	945 (33.1)	< 0.001^++^
Dyslipidemia	947 (32.0)	35 (32.1)	912 (32.0)	1.000
Diabetes mellitus	420 (14.2)	19 (17.4)	401 (14.1)	0.396
Asthma	89 (3.0)	6 (5.5)	83 (2.9)	0.135
Sinusitis	191 (6.5)	6 (5.5)	185 (6.5)	0.832
Allergic rhinitis	424 (14.3)	9 (8.3)	415 (14.6)	0.089
Obstructive sleep apnea	35 (1.2)	0 (0.0)	35 (1.2)	0.401
Kidney disease	68 (2.3)	0 (0.0)	68 (2.4)	0.121
Atopic dermatitis	50 (1.7)	1 (0.9)	49 (1.7)	0.718
Tympanitis	175 (5.9)	4 (3.7)	171 (6.0)	0.421

* P<0.05,

+ P<0.01,

++ P<0.001

Table 2 categorized participants’ features based on the GAD-7 scores into three groups: normal (GAD 0–4 points), mild (GAD 5–9 points), and moderate/severe (GAD 10–21 points). The prevalence of GAD was 14.0% (414/2,960) among those aged ≥40 years. The severity of GAD was significantly associated with age, sex, drinking problems, a chewing problem, physical activity, including strength exercises, household income, BMI, and chronic comorbidities, such as hypertension, sinusitis, and atopic dermatitis.

**Table 2:** Features of study sample based on the severity of generalized anxiety disorder (n = 2,960).

Variables	Overall (n = 2960)	No GAD (n = 2546)	Mild GAD (n = 295)	Moderate & Severe GAD (n = 119)	P-value
Age (years)	59.8 ± 11.6	60.3 ± 11.5	56.2 ± 11.3	59.2 ± 12.6	< 0.001^++^
Male	1,297 (43.8)	1,147 (45.1)	112 (38.0)	38 (31.9)	0.002^+^
Ever married (vs. others)	2,809 (94.9)	2,422 (95.1)	275 (93.2)	112 (94.1)	0.342
Currently smoker (vs. others)	439 (14.8)	381 (15.0)	43 (14.6)	15 (12.6)	0.772
Drinking problem	374 (12.6)	302 (11.9)	52 (17.6)	20 (16.8)	0.007^+^
Chewing problem	1,092 (36.9)	890 (35.0)	137 (46.4)	65 (54.6)	< 0.001^++^
**Physical activity (per week)**					
Walking exercises	2,015 (68.1)	1,740 (68.3)	200 (67.8)	75 (63.0)	0.475
Strength exercises	529 (17.9)	474 (18.6)	41 (13.9)	14 (11.8)	0.028^*^
**Education level**					0.343
Elementary school or below	544 (18.4)	480 (18.9)	41 (13.9)	23 (19.3)	
Middle or high school	1,279 (43.2)	1,094 (43.0)	133 (45.1)	52 (43.7)	
College or above	1,137 (38.4)	972 (38.2)	121 (41.0)	44 (37.0)	
**Household income (quintile)**					0.039^*^
Upper (1st)	678 (22.9)	586 (23.0)	75 (25.4)	17 (14.3)	
Middle (2nd–4th)	1,820 (61.5)	1,575 (61.9)	170 (57.6)	75 (63.0)	
Lower (5th)	462 (15.6)	385 (15.1)	50 (16.9)	27 (22.7)	
**Body mass index category**					0.034^*^
Underweight	48 (1.6)	36 (1.4)	7 (2.4)	5 (4.2)	
Normal	1,101 (37.2)	933 (36.6)	125 (42.4)	43 (36.1)	
Overweight or obese	1,811 (61.2)	1,577 (61.9)	163 (55.3)	71 (59.7)	
**Comorbidities**					
Hypertension	1,000 (33.8)	882 (34.6)	83 (28.1)	35 (29.4)	0.048^*^
Dyslipidemia	947 (32.0)	802 (31.5)	105 (35.6)	40 (33.6)	0.336
Diabetes mellitus	420 (14.2)	359 (14.1)	38 (12.9)	23 (19.3)	0.222
Asthma	89 (3.0)	73 (2.9)	13 (4.4)	3 (2.5)	0.307
Sinusitis	191 (6.5)	155 (6.1)	22 (7.5)	14 (11.8)	0.037^*^
Allergic rhinitis	424 (14.3)	356 (14.0)	48 (16.3)	20 (16.8)	0.417
Obstructive sleep apnea	35 (1.2)	30 (1.2)	3 (1.0)	2 (1.7)	0.846
Kidney disease	68 (2.3)	56 (2.2)	9 (3.1)	3 (2.5)	0.668
Atopic dermatitis	50 (1.7)	37 (1.5)	10 (3.4)	3 (2.5)	0.029^*^
Tympanitis	175 (5.9)	145 (5.7)	20 (6.8)	10 (8.4)	0.379

* P<0.05,

+ P<0.01,

++ P<0.001

Table 3 shows the relationship between sarcopenia, possible sarcopenia, and GAD. Among all participants, sarcopenia and possible sarcopenia correlated with GAD severity in the unadjusted model. The regression coefficients of sarcopenia and possible sarcopenia were 0.137 (95% CI: 0.046–0.228) and 0.069 (95% CI: 0.001–0.136). After adjusting for sex, marital status, smoking, physical activity, and chewing problems (Model 3), sarcopenia was still associated with GAD (P > 0.05). When stratified based on sex, no significant results were found for sarcopenia or possible sarcopenia in males. However, in females, after adjusting for sex, marital status, and smoking (Model 1), sarcopenia and possible sarcopenia showed correlations of 0.145 (95% CI: 0.025–0.266) and 0.096 (95% CI: 0.004–0.189), respectively, with GAD. After further adjustment for physical activity variables (Model 2), only sarcopenia was associated with GAD in females (β=0.132, P = 0.033).

**Table 3:** Multiple linear regression model of sarcopenia, possible sarcopenia, and generalized anxiety disorder.

Models	Sarcopenia (n = 110) β(95% CI)	P-value	Possible sarcopenia (n = 208) β(95% CI)	P-value
Overall (n = 2960)				
Unadjusted	0.137 (0.046, 0.228)	0.003^++^	0.069 (0.001, 0.136)	0.047^*^
Model 1	0.131 (0.040, 0.222)	0.005^++^	0.065 (−0.003, 0.132)	0.061
Model 2	0.126 (0.035, 0.217)	0.007^++^	0.060 (−0.008, 0.128)	0.083
Model 3	0.114 (0.021, 0.207)	0.016^*^	0.051 (−0.017, 0.119)	0.145
Male (n = 1297)				
Unadjusted	0.101 (−0.0386, 0.241)	0.156	0.012 (−0.087, 0.110)	0.818
Model 1	0.102 (−0.037, 0.242)	0.151	0.013 (−0.086, 0.112)	0.797
Model 2	0.103 (−0.037, 0.243)	0.148	0.011 (−0.088, 0.110)	0.826
Model 3	0.102 (−0.040, 0.244)	0.157	0.010 (−0.090, 0.110)	0.844
Female (n = 1663)				
Unadjusted	0.146 (0.025, 0.267)	0.018^*^	0.097 (0.005, 0.189)	0.040^*^
Model 1	0.145 (0.025, 0.266)	0.018^*^	0.096 (0.004, 0.189)	0.041^*^
Model 2	0.132 (0.011, 0.253)	0.033^*^	0.087 (−0.006, 0.179)	0.066
Model 3	0.109 (−0.014, 0.232)	0.083	0.072 (−0.020, 0.165)	0.125

Model 1 = adjusted for sex, marital status, and smoking

Model 2 = adjusted for Model 1 confounders + physical activity

Model 3 = adjusted for model 2 confounders + chewing problems + BMI + chronic diseases.

* P<0.05,

+ P<0.01,

++ P<0.001

Table 4 shows the results of the analysis of the association of sarcopenia with GAD severity. Among all participants, individuals with sarcopenia had a 1.051 times higher probability of experiencing mild GAD than those without sarcopenia (95% CI: 0.554–1.992). Furthermore, individuals with sarcopenia had a 2.480 times higher probability of experiencing moderate and severe GAD than those without sarcopenia (95% CI: 1.232–4.990), and this distinction was significant.

**Table 4:** Association of sarcopenia with severity of generalized anxiety disorder.

Variables	Generalized Anxiety Disorder scores						P-trend
	No GAD		Mild GAD		Moderate & Severe GAD		
	N	OR (95% CI)	N	OR (95% CI)	N	OR (95% CI)	
Unadjusted	2,546	1	295	1.213 (0.655, 2.247)	119	2.913 (1.511, 5.617)	0.0165^*^
Model 1		1		1.184 (0.639, 2.197)		2.783 (1.440, 5.380)	0.003^+^
Model 2		1		1.163 (0.626, 2.160)		2.642 (1.362, 5.127)	0.005^+^
Model 3		1		1.051 (0.554, 1.992)		2.480 (1.232, 4.990)	<0.001^++^

Model 1 = adjusted for sex, marital status, and smoking

Model 2 = adjusted for Model 1 confounders + physical activity

Model 3 = adjusted for model 2 confounders + chewing problems + BMI + chronic diseases.

* P<0.05,

+ P<0.01,

++ P<0.001

GAD, generalized anxiety disorder; OR, odds ratio; CI, confidence interval

## Discussion

Sarcopenia, the age-related loss of muscle mass and strength, significantly impacts overall health, contributing to both physical and psychological burdens [1]. Existing literature highlights the dual impact of sarcopenia on both physical and mental health, linking it to conditions such as generalized anxiety disorder (GAD) [17]. Despite its well-established role in physical rehabilitation, its relevance in psychiatric disorders, particularly GAD, has been largely overlooked [15]. Recent studies suggest that sarcopenia may play a more critical role in mental health than previously recognized, emphasizing the need for further research in this area [7]. Addressing sarcopenia through targeted interventions could enhance both physical and psychological well-being in middle-aged and older adults [6].

This study, using data from the 2022 KNHANES, a large-scale representative survey of the Korean population, is the first to explore the link between sarcopenia and GAD among Korean middle-aged and older adults. Using the AWGS 2019 algorithm, we found that participants with sarcopenia had a higher probability of developing GAD than those without sarcopenia. Therefore, patients with decreased muscle mass and grip strength had higher GAD-7 scores. These findings are consistent with those of other cross-sectional studies, suggesting a close correlation between lower muscle strength and GAD. A study in Ireland involving 3,952 individuals aged >50 years reported that an increase in grip strength was associated with a decrease in GAD [17]. However, recent observational studies examining the connections between sarcopenia and GAD reported variable results. A recent study of 113 patients undergoing hemodialysis found an inverse correlation between muscle loss and increased anxiety scores [20]. Additionally, a study focusing on patients with inflammatory bowel diseases found that sarcopenia, severe anxiety, and elevated C-reactive protein levels were significantly associated with severe fatigue, further emphasizing the complex interplay between physical and psychological health factors [21]. These results underline the importance of addressing sarcopenia and GAD in clinical settings to improve overall patient outcomes for various diseases. Conversely, an analysis of 237 patients with cancer showed no relationship between muscle mass and anxiety distress scores [22]. These differences could arise from the different diagnostic criteria for sarcopenia and GAD. Notably, GAD has several diagnostic options available, including the Beck Anxiety Inventory, State-Trait Anxiety Inventory, Hospital Anxiety and Depression Scale, Hamilton Anxiety Rating Scale, and GAD-7 scale [23]. The GAD-7 scale was used in this study to explore the correlation between sarcopenia and GAD.

When investigating the association of sarcopenia, possible sarcopenia, and GAD scores based on sex, we found that sarcopenia and possible sarcopenia were strongly correlated with GAD in females. Conversely, an insignificant correlation was detected in males, which could be attributed to the small sample sizes. Additionally, females usually have a lower muscle mass than males [24], and there is evidence indicating that this lower muscle mass increases at the onset of menopause, typically at the age of 40 years [25]. Following menopause, reduced estrogen levels have been linked to decreased muscle mass and function [25]. Hormone levels decline with age, particularly sex hormones, which are pivotal in the development of age-related sarcopenia [26]. Furthermore, decreased sex hormones, particularly estrogen, are associated with an increased risk of depression and anxiety disorders in females [27]. Therefore, the relationship between sarcopenia and GAD may be further influenced by declining sex hormone levels, indicating the need to consider the impact of sex differences on GAD with sarcopenia.

The mechanisms underlying the association of sarcopenia with GAD remain largely unclear; however, several hypotheses have been proposed. First, inflammation is a common underlying mechanism of both sarcopenia and GAD. Pro-inflammatory cytokines such as tumor necrosis factor-α and interleukin-6 can cause chronic low-grade inflammation, which contributes to the development of sarcopenia and the pathogenesis of GAD [11]. Second, high oxidative stress levels associated with chronic diseases can result in muscle atrophy and loss [28]. This heightened oxidative stress also significantly contributes to various psychiatric disorders, including GAD [12]. Third, BDNF, a neurotrophin associated with stress-related conditions such as anxiety disorders, is lower in individuals with anxiety disorders than in those without [13]. Considering that skeletal muscle contractions release neurotrophic factors, such as BDNF [14], it can be assumed that these contractions are related to anxiety disorders.

Additionally, emerging evidence suggests that sarcopenia is an often-overlooked risk factor for mental health conditions, particularly in populations vulnerable to both physical and psychological decline [10]. Given the shared pathophysiological pathways between sarcopenia and psychiatric disorders, including chronic inflammation, oxidative stress, and neurotrophic dysregulation [11], further studies are required to determine the exact mechanisms linking muscle loss to anxiety disorders.

Furthermore, sarcopenia and GAD share common lifestyle risk factors, such as lack of physical activity and inadequate nutritional intake, which can exacerbate muscle loss [15]. Given these shared risk factors, interventions targeting inflammation and neurotrophic pathways may have therapeutic implications for both sarcopenia and GAD. Regular physical activity, particularly resistance training, has been shown to reduce systemic inflammation and enhance BDNF levels, contributing to both muscle health and mental well-being [29]. Additionally, dietary strategies such as omega-3 fatty acid supplementation and polyphenol-rich diets have demonstrated anti-inflammatory effects, which may help mitigate sarcopenia progression and anxiety symptoms [30,31]. These findings highlight the need for an integrative approach that incorporates both physical and mental health strategies in clinical practice. Physical activity keeps the blood-brain barrier healthy and functioning by boosting the body’s defense against damage, reducing oxidative stress, and providing anti-inflammatory elements [32]. Specifically, resistance training elevates BDNF levels, potentially preventing anxiety, mitigating age-related skeletal muscle degeneration, and improving muscle strength [29].

However, this study has some limitations. First, the 2022 KNHANES lacked information on physical performance. According to the AWGS 2019 recommendations, diagnosing sarcopenia involves evaluating muscle mass, strength, and/or physical performance. Additionally, possible sarcopenia can be diagnosed based on low muscle strength or physical performance. Consequently, this limitation may have resulted in inaccuracies in identifying participants with possible sarcopenia or sarcopenia. Second, this study’s cross-sectional design restricts our ability to establish the temporal relationship between sarcopenia and GAD. While our findings indicate a significant association between sarcopenia and GAD, the directionality of this relationship remains uncertain. It is possible that sarcopenia contributes to GAD through physiological mechanisms such as inflammation and neurotrophic dysregulation. Conversely, GAD may also lead to muscle loss due to reduced physical activity and chronic stress-related catabolic effects. Furthermore, while some associations in our study exhibited borderline statistical significance, we employed a predefined statistical approach and applied multivariate regression models to adjust for confounders. We adhered to robust statistical practices, including predefined analytical models and comprehensive adjustments for potential confounders, to ensure the validity of our findings. Regardless of statistical significance, all outcomes are transparently reported to maintain objectivity and reproducibility in future research. Given these considerations, our findings should be interpreted with caution, and further longitudinal studies are required to confirm these associations and establish a clearer causal relationship. Although our findings support a significant association between sarcopenia and GAD, we acknowledge that these results should be interpreted cautiously. Future longitudinal and interventional studies are necessary to confirm causality and explore the underlying mechanisms in greater depth. Lastly, this study relied on observational data, which may have been subject to bias owing to confounding factors. Efforts were made to consider as many variables as possible to mitigate these biases. However, the potential influence of other confounders, such as nutritional status, sleep patterns, and dietary habits, cannot be entirely eliminated. Finally, due to sociocultural influences, Koreans may subconsciously respond negatively to questions about mental health [33], potentially leading to an underestimated prevalence of depressive symptoms.

Despite these limitations, this study had several strengths. First, it utilizes extensive and rigorously examined national data, which enhances the generalizability of the findings and statistical reliability. Second, we measured muscle mass using bioelectrical impedance analysis, which enabled us to evaluate individuals with sarcopenia more precisely. Additionally, this study is the first to explore the relationship between sarcopenia and GAD severity. This can influence further academic research and healthcare strategies and raise public awareness of the interconnection between physical and mental health, especially in an aging population.

As sarcopenia significantly affects these areas, targeted interventions to maintain and improve muscle function can enhance both physical and psychological health [6]. Thus, addressing sarcopenia in clinical settings is vital for optimizing recovery and overall well-being in aging populations.

## Conclusions

This study identified the association between sarcopenia and GAD among middle-aged and older adults and established sarcopenia as a potential risk factor for GAD. Early prevention and treatment of sarcopenia may help prevent or delay the onset of GAD in this population, thereby contributing to healthy aging. Understanding the connection between sarcopenia and GAD underscores the importance of a holistic approach to improving the well-being of middle-aged and older adults. Furthermore, this study highlights the complex relationship between physical activity and mental health. Individuals with GAD may be less likely to engage in regular physical activity, which in turn can contribute to further anxiety symptoms. Given this bidirectional relationship, integrating both strength training and mental health strategies into health programs may be beneficial. This study also encourages healthcare professionals to consider the link between muscle strength and GAD when caring for older adults and advocate for integrated assessments and interventions. Additional longitudinal studies are essential to comprehensively understand the relationship between sarcopenia and GAD. Such research could track changes over time and provide further insight into how the progression of sarcopenia affects the development of GAD. Future research should also consider other factors, such as sleep patterns and nutritional status, which play crucial roles in managing both sarcopenia and GAD. Additionally, it is necessary to investigate cultural factors that influence the diagnosis and treatment of GAD, especially in Koreans. A deeper understanding of these cultural aspects will help develop more effective, culturally tailored prevention strategies.
